# Supplementary material for: Exploring the use of art interventions in challenging stigmas related to neurological disorders: A scoping review
Source: PLoS One. 2026 Mar 27;21(3):e0328317. doi: 10.1371/journal.pone.0328317 (PMC13029808; doi:10.1371/journal.pone.0328317)
Supplement: S2 File — (DOCX) [file pone.0328317.s002.docx]

**S1 Appendix. Search strategy**

(Artistic OR artistry OR creative* OR performance* OR “literacy” OR visual* OR film* OR video* OR dance* OR “theater” OR “theatre” OR “visual reality” OR paint* OR draw* OR “drama” OR narrative* OR comic* OR poetry*) AND (Neurological disorder* OR neurological condition* OR brain disorder* OR Parkinson* OR epilepsy* OR dementia* OR neurodegenerative disorder* OR cognitive impairment* OR cognitive function* Or stroke*) AND (discriminate* OR social stigma* OR stereotype* OR marginalize* OR marginalise* OR negative perception* OR dehumanize* OR dehumanise* OR bias* OR Awareness* OR educate* OR knowledge* OR sensitize* OR promote* OR advocate* OR “community education” OR “perception” OR “engagement” OR “public outreach” OR campaign*)
