# Supplementary material for: Exploring the use of art interventions in challenging stigmas related to neurological disorders: A scoping review
Source: PLoS One. 2026 Mar 27;21(3):e0328317. doi: 10.1371/journal.pone.0328317 (PMC13029808; doi:10.1371/journal.pone.0328317)
Supplement: Attachment — Submitted filename: Review.pdf [file pone.0328317.s004.pdf]

Reviewer

The authors present a review investigating Exploring the use of art interventions in challenging stigmas related to neurological disorders: A scoping review. The study is well-structured and addresses an important topic. I have a few comments and suggestions that could help further improve the clarity of the manuscript.

Comments:

Introduction

1. Line 69-70 the authors mentioned “These stigmas are reinforced by cultural beliefs, fear, and the visibility or unpredictability of symptoms”. The authors may consider highlighting whether certain cultural stigmas related to neurological disorders are shared across countries with different income levels.

2. Line 70-71 The article mentions that disorders like epilepsy or Parkinson’s are sometimes perceived as spiritual afflictions or witchcraft, fueling rejection. Are beliefs that disorders such as epilepsy or Parkinson’s are spiritual afflictions or witchcraft primarily reported in African countries, or do similar perceptions occur in other regions as well?

3. The authors may also consider adding brief context on how cultural stigmas influence both access to healthcare and the social inclusion of individuals with neurological disorders in different settings.

Methods

4. Section 2.2 - The authors mention that studies were reviewed up to October 2024. It would be helpful to indicate the starting date to clarify the full-time span of the literature considered.

5. Section 2.5 – The authors mention that a data extraction form was developed to collect relevant information. Could you consider making this form publicly available, as it could help other research groups conduct similar reviews?

Results

6. In Section 3.5, the authors describe the number of art sessions and outcomes for participants (reference 36). It would be helpful to clarify whether the reviewed studies reported the duration of each intervention, and if so, to indicate this information, as it could provide important context for interpreting the results.

Discussion

7. The authors report in the abstract that most studies were conducted in high-income settings. It would be valuable to discuss whether this pattern reflects a higher prevalence of neurodegenerative diseases in these regions or is predominantly driven by the greater availability of research resources in high-income countries.

8. Given that most of the cited studies did not examine gender, it could be valuable to consider hypotheses regarding how gender-related factors might influence interest or engagement in art.

9. The authors could consider discussing the hypothesis that inclusive family environments support continued engagement in art-based interventions, potentially enhancing long-term quality of life.
