# Supplementary material for: Exploring the use of art interventions in challenging stigmas related to neurological disorders: A scoping review
Source: PLoS One. 2026 Mar 27;21(3):e0328317. doi: 10.1371/journal.pone.0328317 (PMC13029808; doi:10.1371/journal.pone.0328317)
Supplement: Attachment — Submitted filename: Response to reviewers.docx [file pone.0328317.s006.docx]

**Response to reviewers**

Thank you for considering this manuscript for publication within PLOS One. The reviewer comments have been incredibly insightful for improving the quality of this manuscript. Please see below for a list of reviewer feedback and the changes made to the manuscript based upon this.

Update to the financial disclosure statement: “This project was funded by the British Academy grant number OIIRP230192. The funder had no role in the study design, analysis, decision to publish, or preparation of the manuscript.”

Kind regards

Mary A. Bitta

On behalf of all authors

**Journal Requirements:**

When submitting your revision, we need you to address these additional requirements.

1. Please ensure that your manuscript meets PLOS ONE's style requirements, including those for file naming. The PLOS ONE style templates can be found at

[https://journals.plos.org/plosone/s/file?id=wjVg/PLOSOne_formatting_sample_main_body.pdf](https://eur03.safelinks.protection.outlook.com/?url=https%3A%2F%2Fjournals.plos.org%2Fplosone%2Fs%2Ffile%3Fid%3DwjVg%2FPLOSOne_formatting_sample_main_body.pdf&data=05%7C02%7CJ.Lumsdon2%40newcastle.ac.uk%7C56969d07cd374254a1ed08dde08d7af4%7C9c5012c9b61644c2a91766814fbe3e87%7C1%7C0%7C638913620434138424%7CUnknown%7CTWFpbGZsb3d8eyJFbXB0eU1hcGkiOnRydWUsIlYiOiIwLjAuMDAwMCIsIlAiOiJXaW4zMiIsIkFOIjoiTWFpbCIsIldUIjoyfQ%3D%3D%7C0%7C%7C%7C&sdata=LgaalWLGLTxtmb3Gbt%2B4dDEw3979m9eLlxLjZeStqjo%3D&reserved=0) and

[https://journals.plos.org/plosone/s/file?id=ba62/PLOSOne_formatting_sample_title_authors_affiliations.pdf](https://eur03.safelinks.protection.outlook.com/?url=https%3A%2F%2Ftrack.editorialmanager.com%2FCL0%2Fhttps%3A%252F%252Fjournals.plos.org%252Fplosone%252Fs%252Ffile%253Fid%3Dba62%252FPLOSOne_formatting_sample_title_authors_affiliations.pdf%2F1%2F010f0198c9db0ac6-124a9a0d-495d-4bfe-80c7-e15b86431779-000000%2Fjuwxk9U0ubBwK6CMJPzXBslOe0QWcdZPFGEQcuodh-g%3D225&data=05%7C02%7CJ.Lumsdon2%40newcastle.ac.uk%7C56969d07cd374254a1ed08dde08d7af4%7C9c5012c9b61644c2a91766814fbe3e87%7C1%7C0%7C638913620434152949%7CUnknown%7CTWFpbGZsb3d8eyJFbXB0eU1hcGkiOnRydWUsIlYiOiIwLjAuMDAwMCIsIlAiOiJXaW4zMiIsIkFOIjoiTWFpbCIsIldUIjoyfQ%3D%3D%7C0%7C%7C%7C&sdata=hIHHEJADCSJzwS7%2BJPA38mp%2BcYRzrkCaL92snv71FTM%3D&reserved=0).

**Response:** **Updated**

2. Thank you for stating the following financial disclosure:

“This project was funded by the British Academy grant number OIIRP230192”

Please state what role the funders took in the study.  If the funders had no role, please state: "The funders had no role in study design, data collection and analysis, decision to publish, or preparation of the manuscript."

If this statement is not correct you must amend it as needed.

Please include this amended Role of Funder statement in your cover letter; we will change the online submission form on your behalf.

**Response:** **Amended the statement and included in the above cover letter**

3. We note that your Data Availability Statement is currently as follows: [All relevant data are within the manuscript and its Supporting Information files.]

Please confirm at this time whether or not your submission contains all raw data required to replicate the results of your study. Authors must share the “minimal data set” for their submission. PLOS defines the minimal data set to consist of the data required to replicate all study findings reported in the article, as well as related metadata and methods ([https://journals.plos.org/plosone/s/data-availability#loc-minimal-data-set-definition](https://eur03.safelinks.protection.outlook.com/?url=https%3A%2F%2Fjournals.plos.org%2Fplosone%2Fs%2Fdata-availability%23loc-minimal-data-set-definition&data=05%7C02%7CJ.Lumsdon2%40newcastle.ac.uk%7C56969d07cd374254a1ed08dde08d7af4%7C9c5012c9b61644c2a91766814fbe3e87%7C1%7C0%7C638913620434164914%7CUnknown%7CTWFpbGZsb3d8eyJFbXB0eU1hcGkiOnRydWUsIlYiOiIwLjAuMDAwMCIsIlAiOiJXaW4zMiIsIkFOIjoiTWFpbCIsIldUIjoyfQ%3D%3D%7C0%7C%7C%7C&sdata=3rWOuOMeqNZeZFJEHtOOt4565Qyu5a2AdDFyaCoemiY%3D&reserved=0)).

For example, authors should submit the following data:

- The values behind the means, standard deviations and other measures reported;

- The values used to build graphs;

- The points extracted from images for analysis.

Authors do not need to submit their entire data set if only a portion of the data was used in the reported study.

If your submission does not contain these data, please either upload them as Supporting Information files or deposit them to a stable, public repository and provide us with the relevant URLs, DOIs, or accession numbers. For a list of recommended repositories, please see [https://journals.plos.org/plosone/s/recommended-repositories](https://eur03.safelinks.protection.outlook.com/?url=https%3A%2F%2Fjournals.plos.org%2Fplosone%2Fs%2Frecommended-repositories&data=05%7C02%7CJ.Lumsdon2%40newcastle.ac.uk%7C56969d07cd374254a1ed08dde08d7af4%7C9c5012c9b61644c2a91766814fbe3e87%7C1%7C0%7C638913620434179814%7CUnknown%7CTWFpbGZsb3d8eyJFbXB0eU1hcGkiOnRydWUsIlYiOiIwLjAuMDAwMCIsIlAiOiJXaW4zMiIsIkFOIjoiTWFpbCIsIldUIjoyfQ%3D%3D%7C0%7C%7C%7C&sdata=MMMgFY%2BKSzQrUTPxKjFWwF4qdLWVl%2FpQtBBcJ7ebFzQ%3D&reserved=0).

If there are ethical or legal restrictions on sharing a de-identified data set, please explain them in detail (e.g., data contain potentially sensitive information, data are owned by a third-party organization, etc.) and who has imposed them (e.g., an ethics committee). Please also provide contact information for a data access committee, ethics committee, or other institutional body to which data requests may be sent. If data are owned by a third party, please indicate how others may request data access.

**Response:**

**As this manuscript is a review, no data has been generated, although the tables contain all necessary data needed to replicate the analysis of included studies**

4. Please include captions for your Supporting Information files at the end of your manuscript, and update any in-text citations to match accordingly. Please see our Supporting Information guidelines for more information: [http://journals.plos.org/plosone/s/supporting-information](https://eur03.safelinks.protection.outlook.com/?url=http%3A%2F%2Fjournals.plos.org%2Fplosone%2Fs%2Fsupporting-information&data=05%7C02%7CJ.Lumsdon2%40newcastle.ac.uk%7C56969d07cd374254a1ed08dde08d7af4%7C9c5012c9b61644c2a91766814fbe3e87%7C1%7C0%7C638913620434190440%7CUnknown%7CTWFpbGZsb3d8eyJFbXB0eU1hcGkiOnRydWUsIlYiOiIwLjAuMDAwMCIsIlAiOiJXaW4zMiIsIkFOIjoiTWFpbCIsIldUIjoyfQ%3D%3D%7C0%7C%7C%7C&sdata=Zq64osboNKDXJEUibXsRRq4YbN7W9%2BUaGze3Olr%2BORs%3D&reserved=0). **U**

**Response: Updated the formatting of the manuscript**

5. If the reviewer comments include a recommendation to cite specific previously published works, please review and evaluate these publications to determine whether they are relevant and should be cited. There is no requirement to cite these works unless the editor has indicated otherwise.

**Response:** **Reviewer #2 suggested the inclusion of two studies within the review, these are both appropriate studies which would have been included had they appeared within the search, hence, they have been included within the review.**

6. Please review your reference list to ensure that it is complete and correct. If you have cited papers that have been retracted, please include the rationale for doing so in the manuscript text, or remove these references and replace them with relevant current references. Any changes to the reference list should be mentioned in the rebuttal letter that accompanies your revised manuscript. If you need to cite a retracted article, indicate the article’s retracted status in the References list and also include a citation and full reference for the retraction notice.

**Response:** **Reviewed to ensure references are complete and correct. Several additional references have been added following changes to the manuscript.**

**Reviewer #1:** This manuscript effectively addresses how lack of knowledge and misconceptions about neurological disorders often foster prejudice, such as the perception that “people with abnormal movements = people with mental illness.” The authors’ findings suggest that educational and awareness activities can shift this perception toward “people with abnormal movements = patients.” From the results presented, the following sequence of cognitive change can be inferred:
An initial recognition that the individual has a medical condition.
Activation of the moral value that it is wrong to hold negative views toward people who are ill.
Based on this moral value, acquisition of knowledge about the disease leads to a reduction in stigma.

In contrast, in low- and middle-income countries, several factors may contribute to the persistence of stigma: (i) lack of awareness about neurological diseases due to limited medical and educational resources, (ii) insufficient development of the moral framework that underpins non-discriminatory attitudes toward illness, and (iii) a high prevalence of untreated patients whose symptoms remain pronounced in daily life. A deeper analysis of these specific circumstances could strengthen the manuscript’s discussion of challenges unique to low- and middle-income settings.

**Response: Points suggested help to further elaborate on the context in which stigma occurs, this has been incorporated into the second paragraph of the introduction and the final paragraph of the key findings section in the discussion to further highlight the unique factors which influence the experience of stigma in low- and middle-income countries.**

Furthermore, the background and discussion presented by the authors hold important implications in the context of global migration and labor mobility, where cultural and religious backgrounds are increasingly diverse. For example, when migration limits access to religious institutions or community networks, the continuity of moral education and shared values may be disrupted, potentially hindering cross-cultural understanding of disease and the reduction of stigma. Through the framework provided by this study, exploring the relationship between changes in moral education, cultural background, and stigma could offer valuable—albeit delicate—insights from both medical and societal perspectives. Therefore, the significance of this work lies not only in evaluating the effectiveness of art-based interventions but also in raising awareness of underlying issues related to moral education, cultural foundations, and the transformation of values in the context of international migration—issues that are contemporary, unavoidable, and worth understanding through a medical lens.

**Response:** **Really insightful point around the complexity of stigma and the multiple perspectives needed to be considered when designing interventions. Unfortunately, only a single article explored the culturally tailoring an intervention and did not provide any details about the process of tailoring an intervention to different cultural and religious backgrounds and so it is difficult to elaborate upon this point – this is mentioned in the benefits and challenges to art-based interventions section. Considering LMICs are most in need of anti-stigma interventions, further details on this point have been elaborated in the discussion at the end of the key findings section to give further context.**

**Reviewer #2:** This is an interesting paper that summarizes the art-based interventions reported in literature to tackle stigma in neurological disorders.
It is a nice ad useful review that helps to give clarity and consistence to a delicate and under-valued topic.
I will try to suggest some revisions that might improve the paper.

Terminology:
•Page 5 line 55: insert (LMIC) next to the extended expression **Added**
•Page 6 lines 93-94: delete the sentence: One such approach is the use of art to challenge negative stereotypes and foster social change. (repetition)

**Response:** **Deleted the final sentence of the previous paragraph**
•Page 7 line 105 delete “the” before performing arts **Deleted**

Concept
I was surprised to notice the paucity of data about Africa and South America. I found that some interesting papers have not been cited in your revision. For example, a comic-book based study in Bolivia (Cicero CE, et al. Comic book-based educational program on epilepsy for high-school students: Results from a pilot study in the Gran Chaco region, Bolivia. Epilepsy Behav. 2020 Jun; 107:107076. doi: 10.1016/j.yebeh.2020.107076. Epub 2020 Apr 18. PMID: 32315969.) and one study among students in Madagascar (Mioramalala SA, et al. Effects of an educational comic book on epilepsy-related knowledge, attitudes and practices among schoolchildren in Madagascar. Epilepsy Res. 2021 Oct;176:106737. doi: 10.1016/j.eplepsyres.2021.106737. Epub 2021 Aug 9. PMID: 34419769) Did you exclude these articles on purpose, and, in case, for which reasons, or your search strategy was not able to find these papers?

**Response:** **Thank you for recommending these studies, they are both extremely relevant to our review, hence they have both been added into the review and the results section updated as appropriate. Unfortunately, our search strategy was not able to find these articles and so thank you for bringing these to our attention.**

**Reviewer #3:** The manuscript provides a well-structured review with a clear summary of the literature. I have provided comments and suggestions for improvement; overall, the review represents a valuable contribution and could benefit from minor clarifications and further discussion of future research directions.

The authors present a review investigating Exploring the use of art interventions in challenging stigmas related to neurological disorders: A scoping review. The study is well-structured and addresses an important topic. I have a few comments and suggestions that could help further improve the clarity of the manuscript.

**Introduction**

Line 69-70 the authors mentioned “These stigmas are reinforced by cultural beliefs, fear, and the visibility or unpredictability of symptoms”. The authors may consider highlighting whether certain cultural stigmas related to neurological disorders are shared across countries with different income levels.

**Response: Thank you for your comments, additional details around shared cultural stigmas across different contexts have been added into this paragraph to better improve the generalisability of this review**

2. Line 70-71 The article mentions that disorders like epilepsy or Parkinson’s are sometimes perceived as spiritual afflictions or witchcraft, fueling rejection. Are beliefs that disorders such as epilepsy or Parkinson’s are spiritual afflictions or witchcraft primarily reported in African countries, or do similar perceptions occur in other regions as well?

**Response:** **Clarifications have been added that beliefs around witchcraft and curses are specific to Africa and added in that other continents such as Asia struggle with other forms of cultural specific stigmas such as secretiveness around diagnoses.**

3. The authors may also consider adding brief context on how cultural stigmas influence both access to healthcare and the social inclusion of individuals with neurological disorders in different settings.

**Response:** **Rounded out the paragraph to tie in different cultural stigmas around neurological disorders and how that impacts healthcare and wellbeing.**

**Methods**

4. Section 2.2 - The authors mention that studies were reviewed up to October 2024. It would be helpful to indicate the starting date to clarify the full-time span of the literature considered.

**Response: Clarified that articles were searched from inception to October 2024**

5. Section 2.5 – The authors mention that a data extraction form was developed to collect relevant information. Could you consider making this form publicly available, as it could help other research groups conduct similar reviews?

**Response: Thank you for your suggestion, as the data extraction form was simplified and streamlined to form Table 1. It seems redundant to also include the data extraction form as it includes many of the same details**

**Results**

6. In Section 3.5, the authors describe the number of art sessions and outcomes for participants (reference 36). It would be helpful to clarify whether the reviewed studies reported the duration of each intervention, and if so, to indicate this information, as it could provide important context for interpreting the results.

**Response**: **Data on intervention duration was extracted and is presented in Table 1 but agreed that this should be made clearer. I have written a sentence about intervention duration at the beginning of the section on impact of art interventions in reducing stigma and mechanisms of change and have included details around intervention duration where relevant throughout the results section.**

**Discussion**

7. The authors report in the abstract that most studies were conducted in highincome settings. It would be valuable to discuss whether this pattern reflects a higher prevalence of neurodegenerative diseases in these regions or is predominantly driven by the greater availability of research resources in highincome countries.

**Response:** **Prevalence of neurological disorders remains the same if not higher in lower- and middle-income countries, although this is commonly under reported due to poor methodological quality of prevalence studies. Most likely, fewer studies were conducted in lower- middle-income countries due to poorer availability of research resources and reduced priority of stigma interventions. This has been added into the final paragraph in they key findings section of the discussion to give further context.**

8. Given that most of the cited studies did not examine gender, it could be valuable to consider hypotheses regarding how gender-related factors might influence interest or engagement in art.

**Response**: **Gender dynamics were poorly reported on in included studies which hindered the ability to discuss this within the review, future directions have now been included around examining how gender related factors may influence the efficacy of art interventions on stigma**

9. The authors could consider discussing the hypothesis that inclusive family environments support continued engagement in art-based interventions, potentially enhancing long-term quality of life.

**Response:** **Family support structures was not a strong theme within the included articles so it would be difficult to adequately address this point with the available data although this is an interesting point that should be explored in future research**
